# Dr. Jno. T. Sharpless’s Case of Preservation of the Human Body by the Acetate of Alumina

**Published:** 1842-08-13

**Authors:** Jno. T. Sharpless

**Affiliations:** Philadelphia


### MEDICAL EXAMINER.

NEW SERIES.

No. 33.] PHILADELPHIA, AUGUST 13, 1842. [Vol. I.

### *A Case of Preservation of the Human Body by the Acetate of Alumina.*

By Jno. T. Sharpless, M. D., Philadelphia.

As the process of embalming the human body, for the purpose of transportation or long preservation has rarely been attempted in this city, (except for the purposes of the Anatomist,) it may prove interesting and useful to state the result of an experiment recently made.

A gentleman from Canada, aged 64 years, died in June last of cancer of the stomach. One year previous he weighed 208 pounds, and at his death about 140 pounds, and had become very much infiltrated with water. To enable his family to take him home, Dr. Wm. R. Grant, Demonstrator of Anatomy of Jefferson College, and the writer, undertook to preserve the body. The weather was very sultry, with rain and sun nearly every day.

He died at 6 o'clock A. M. on Friday. In the afternoon the only incision that was permitted by the family, was made in the middle of the abdomen, and large injecting tubes were placed in the aorta below the superior mesenteric artery, one pointing each way. A saturated solution of corrosive sublimate in alcohol was thrown in, a quart upward and a pint downward, and the body was kept damp by a weaker mixture.

We next day injected the same quantity of a saturated solution of the acetate of alumine, which had the immediate effect of giving the whole body a manifest rose colour, making it resemble life in a remarkable degree. The face and hands were kept constantly damp with the solution, and the body frequently washed with it.

The next day, Sunday, being an exceedingly hot day, there was a green stripe the whole length of the fibula of one side, and a similar spot upon the ribs, with vesications on several parts of the body, but no odour or other indications of putrefaction. The usual blue effusion of blood along the back and under parts had changed to a bright red, which remained to the last. We again injected nearly the same quantity of the aluminous solution with sufficient force to distend the arteries of the forehead, during which operation the green stripes upon the leg and ribs changed to a light brown, which colour they retained until the body was sent away. On Monday a pint of the solution was injected, the tubes removed, the aorta well secured, and the abdomen closed, and at 9 P. M. the body was placed in the coffin, with cotton wadding dipped in alcohol around the face and hands. The joints were all perfectly flexible; the skin of the limbs and body was so like life, that, from appearances, no one would have believed them dead, the hands more particularly, the skin of which was very soft, and the fingers, were beautifully tinted with a bright rose colour, as were also the convolutions of the ears. The face was less natural, having shrivelled from the constant contact of the solution. There was no ice used, or any precaution against the heat, the body being in a room with little circulation of air, and the sun shining in and even upon the body several hours in the morning.

It was removed next day, Tuesday. During its transit, it was, of course, exposed to great heat from the sun, the weather remaining exceedingly warm, and the size of the enclosing box preventing it being well covered. It arrived at home on Friday afternoon, the eighth day after death. The appearance upon arrival I will give as received by letter. "The coffin was opened at four o'clock P. M., and you would have been much pleased to have seen how slight a change had taken place; none whatever in the face, unless it was a little thinner. The hand, left exposed for view, was, however, less beautiful than when you saw it, having become, with the exception of the fingers, of a darkish hue. The next day the face began also to change in colour." There was a slight odour, but not of putrefaction, or any other indication of such a change. The body was buried on Monday, the eleventh day after death. The brown colour mentioned was from mere desiccation of the skin, and if the parts had been kept moist, the colour would have remained white and natural.

The sublimate was used in this case to make the result more certain, (although perhaps unnecessary,) the time required for preservation being long, the weather exceedingly warm, and the subject a bad one, from the great infiltration of water through all the cellular membranes. Whether the delicate rose colour, so perceptible upon the surface, arose from the chemical action between the two injected fluids is not known, but it certainly added much to the natural appearance of the body.

